# Lay Misperceptions of Culture as “Biological” and Suggestions for Reducing Them

**DOI:** 10.1177/17456916231181139

**Published:** 2023-07-26

**Authors:** Ronda F. Lo, Joni Y. Sasaki

**Affiliations:** 1 Department of Psychology, York University; 2 Department of Psychology, University of Hawai‘i at Ma-noa

**Keywords:** culture, cultural psychology, cross-cultural psychology, folk biology, biology, lay perceptions

## Abstract

Culture is typically studied as socialized and learned. Yet lay intuitions may hold that culture is associated with biology via perceptions of race, presenting a problem for those who study culture: There may be a mismatch between how psychologists study culture and how their research is interpreted by lay audiences. This article is a call to researchers to recognize this mismatch as a problem and to critically evaluate the way we study culture. We first describe evidence that laypeople tend to associate culture with notions of folk biology. Next, we propose three suggestions for researchers: explicitly address whether biological processes are, or are not, relevant for studying culture in their work; consider using multiple methods because different methods for studying culture may come with assumptions about culture as more tied to socialization or biology; and represent all people as cultural by studying multiple forms of culture and by contextualizing all psychological research. Last, we provide an example for how researchers can implement these suggestions to encourage more accurate interpretations of findings.

As with many topics in psychology, such as love, motivation, and consciousness, culture is something that almost anyone can talk about and yet is not easily defined. Laypeople and scientists alike have many different ideas about what does and does not constitute culture. Whereas scientists’ definitions emphasize that culture is learned, laypeople may assume that culture is based on ancestry or genes—an intuition with psychological roots in essentialism (Medin, 1989) and related biases of thought.^1^ Intuitions that culture is tied to biological makeup can be seen everywhere, from everyday conversations to political rhetoric. For example, when a former United States president tells several American-born, ethnic minority congresswomen to “go back and help fix . . . places from which they came” (Rogers & Fandos, 2019), it exposes an underlying intuition that ancestry or “biology” is more relevant for cultural membership than learned or socialized experiences. In contrast to lay misperceptions of culture as biological, we believe most psychologists intend to study culture as a socialized and learned process. Thus, there may be a mismatch between how scientists study culture in the field^2^ and the way lay audiences interpret cultural research.

We propose that a careful examination of how we study culture may help reduce misperceptions. In the first part of this article, we discuss evidence of these misperceptions of culture as biological. In the second part of this article, we offer three main suggestions for researchers to combat these misperceptions. First, we suggest that explicitly addressing the extent to which actual biological processes are, or are not, relevant for the study of culture in a researcher’s work may reduce audience misinterpretations that observed cultural effects are associated with inaccurate notions of biology (e.g., ancestry and genes). Second, we suggest that researchers consider multiple methods for studying culture. Certain methods make it easier for lay audiences to assume that culture is related to biology (e.g., demographics and ancestry). By providing an overview of common methods used in cultural psychology, we highlight what researchers can learn best from different methods and discuss how lay audiences may come away with different assumptions about culture depending on the method. Third, we suggest that researchers represent all humans as cultural. Known biases show that culture is assumed to be most relevant for groups with non-Western ancestry (i.e., “cultural (mis)attribution bias”; Causadias, Vitriol, & Atkin, 2018). To discourage associating culture with non-Western ancestry, we suggest two ways for researchers to encourage broad representation of people as cultural: study multiple forms of culture (e.g., religion, social class) and contextualize all human psychological work as culturally bound. In the last part of our article, we provide an example for how all researchers can encourage accurate interpretations of their findings. In sum, we offer three main suggestions for researchers who want to incorporate culture in their work:

1. Explicitly address biological processes: Discuss the extent to which biological processes are or are not relevant for the cultural investigation of interest.
2. Consider multiple methods: Anticipate how different methods for studying culture may be interpreted and consider multimethod approaches.
3. Represent all people as cultural: Consider ways in which all people have multiple forms of culture and contextualize psychological research equitably rather than only labeling research about ethnic/racial minorities or non-Western samples as “cultural.”

Many researchers studying culture are already mindful of the issues put forth here. By bringing these ideas together, we hope this article will encourage researchers to grapple with them more explicitly, as well as provide a resource for researchers, educators, and students.

## Laypeople Associate Culture With Folk Biology

Researchers who study culture tend to theorize about culture as predominantly socialized, and they may assume that their lay audience (e.g., those outside of psychology, nonacademic audiences) will also think that culture comes from socialization. However, we propose there are psychological underpinnings that make it difficult for laypeople to resist associating culture with *folk biology* (i.e., folk theories of biology; see Gelman & Legare, 2011). That is, laypeople may have intuitions that culture is associated with folk notions of biology, such as ancestry, genes, or “blood” (e.g., Gil-White, 2001; Waxman et al., 2007). One way this intuition may occur is through perceptions of race. In this section, we first describe empirical evidence that laypeople tend to think of culture as conflated with race. Second, we describe empirical evidence that laypeople tend to believe race is biologically determined. Third, we describe real-world examples that illustrate that perceptions of culture and folk biology seem to be inextricably linked, psychologically and institutionally.

### Perceptions of culture and race are conflated

Perceptions of culture and race may be inextricably linked in the mind such that discussions about culture may bring up notions of race, or vice versa. When laypeople think about culture, they may often think about minorities or foreigners who look visibly different than the majority race. Mentions of culture within a North American context, for example, may bring forth discussions about racial-minority groups (e.g., Asian Americans), whereas discussions of culture within Japan may bring forth discussions about foreigners of a different race (e.g., White Americans). Because racial-minority groups or foreigners in these examples may differ from the majority population in cultural upbringing (culture) and physical appearance (perceived race), laypeople may commonly think about different cultures as linked to different races.

Empirical evidence supports this psychological culture-race link. Both White and Asian American participants, for example, tended to associate Asian faces as less American than White faces (Cheryan & Monin, 2005). Even when researchers used a well-known Asian American celebrity (i.e., Lucy Liu) and White British celebrity (i.e., Kate Winslet), American participants implicitly associated White faces as more American than Asian faces (Devos & Ma, 2008), suggesting that people tend to rely on race to infer national culture. Other studies demonstrate that lay theories of race have downstream consequences for understanding culture. For example, Chinese American participants who endorsed deterministic racial beliefs (e.g., “To a large extent, a person’s race biologically determines his or her abilities and traits”) or who were manipulated into believing race was deterministic (i.e., by reading an article) were more likely to experience greater interference thinking about their Chinese and American culture, talking about their different cultural experiences, and identifying with American culture (Chao et al., 2007; No et al., 2008). These results suggest that perceptions of race can influence and interfere with laypeople’s perceptions of culture, implicating race and culture as linked in some laypeople’s minds.

Historically, the terms “culture,” “race,” and “ethnicity” have been used interchangeably and conflated with each other (Causadias, Vitriol, & Atkin, 2018; Okazaki & Sue, 1995; Quintana et al., 2006), likely in part because of their shared nomological network (Causadias, Vitriol, & Atkin, 2018). Methodologically, the terms “race” and “ethnicity” have also been used as proxies for culture in studies (see Causadias, 2013; Okazaki & Sue, 1995), often through self-reported demographics (see our second suggestion above). This issue has been partly ameliorated as a result of greater education about the differences between culture and race (and ethnicity). Culture is often defined as a collection of socially learned and transmitted values, norms, and behaviors, whereas perceived race tends to be based on physical appearance, and the definition of race changes depending on the social and historical context (Betancourt & López, 1993; Causadias et al., 2023; Smedley & Smedley, 2005). “Ethnicity,” a term sometimes used interchangeably with race, describes membership to a group characterized by common language, customs, and ancestry (Betancourt & López, 1993; Causadias et al., 2023). Although we believe researchers today have a much greater understanding of the differences between culture and race, the history of culture and race being measured in the same way and referred to nondistinctly has likely made it easier for lay audiences to misinterpret research about culture as potentially about race.

### Race is perceived as biological

Although people may not agree on what exactly race is, explicit definitions tend to involve a combination of sociocultural factors (e.g., cultural norms, history) and phenotypic characteristics (e.g., skin tone, physical features; Betancourt & López, 1993; Shih & Sanchez, 2009). Most importantly, racial categories are considered socially and politically constructed and depend on historical power dynamics (Lipsitz, 1998) and the broader social context (J. M. Chen et al., 2018; Helms et al., 2005). There is more genetic variation within racial categories than between, and perceived physical differences between races are due to a very small proportion of genes (Lewontin, 1972; Long & Kittles, 2003), suggesting that genetic variation cannot justify racial categories drawn according to perceived physical differences.

Despite ample evidence that perceived racial categories are not empirically linked to actual biology, laypeople tend to associate race with folk biology, and essentialist thinking may be the psychological root of this association. Essentialist thinking is the view that things in the world have underlying, unseen “essences” that determine what they are versus what they are not (Medin, 1989; Medin & Ortony, 1989). *Biological essentialism* specifically targets notions of ancestry, genes, or blood as the basis for folk biological essences (Keller, 2008). These essentialist beliefs extend to *racial essentialism*, the view that race is determined through folk biological essences. Racial essentialism is both early developing (Gelman, 2003) and found in many different cultural societies (Vezo children in Madagascar: Astuti, 2001; Torguud herders in Mongolia: Gil-White, 2001; urban, middle-class Brazilians: Sousa et al., 2002; Native American Menominee and Americans: Waxman et al., 2007), suggesting that folk biological explanations for race may be found across many different cultural contexts.

### Real-world examples

Given that laypeople tend to conflate culture and race and perceive race as related to folk biology, it is unsurprising that they may also intuitively associate culture with folk biology. Prominent real-world examples can illustrate how laypeople prioritize folk biological intuitions when thinking about culture. For example, laypeople may use folk biological evidence in the form of genes, ancestry, or blood to determine rightful membership to a cultural group. Laypeople may choose to use DNA services that trace ancestry from personal DNA samples (e.g., 23andMe) to reaffirm or “prove” claimed cultural membership or even reconceptualize cultural identity on the basis of newly discovered ancestry in one’s DNA (Marcon et al., 2021; Strand & Källén, 2021). Cultural membership by blood has also been institutionalized. A prominent example includes the U.S. blood quantum laws used to determine who is and is not considered Native American. Many Native American tribes today continue to require some minimum proportion of relatedness to a full-blooded relative using documented lineage, blood quantum, or both, to be formally admitted into a tribe (Schmidt, 2011). These examples illustrate that in the eyes of the public, folk biology is often perceived to be an important determinant of cultural membership to the extent that it has, in some cases, been historically institutionalized.

Laypeople may perceive folk biology as important for the maintenance of culture. Consider, for example, that many Indigenous groups, such as Native Hawaiians (Kānaka^3^ Maoli) and Native Americans, face threats to cultural continuity. The value of cultural continuity is particularly important in Indigenous populations that have experienced, and continue to experience, the effects of colonization and systemic oppression. Indigenous communities have also continued to endure an increasing loss of “full-blooded” members. Given that culture is socialized (i.e., not carried by blood), cultural traditions and values can be transmitted for members regardless of ancestry. These issues of cultural continuity and loss of full-blooded members are both important, and they are also distinct. However, some laypeople may perceive that the increased loss of full-blooded members is necessarily tied to cultural continuity, and there may be complex historical reasons for this lay perception, including conditional rights for Indigenous groups based on colonial blood quantum policies. As Kanaka Maoli anthropologist Kauanui (2002) wrote, “The mixed-race status of Hawaiians [has been used as] both a desired outcome of assimilation, and also a condition that disqualifies them from land rights and other benefits” (p. 119). These blood quantum-based policies for land distribution may have reinforced the idea that cultural identity or authenticity can be proven via percentage of Hawaiian ancestry (for a discussion of blood quantum logic embedded in colonial policies, see Kauanui, 2008). The belief that a part-Hawaiian person would somehow not be “Hawaiian enough” in their cultural identity (e.g., Day, 2005) comes from an American conception of race that historically did not acknowledge multiracial people who may fully embrace multiple identities (e.g., Ledward, 2007). There have been calls to challenge this Americanized discourse that Hawaiian cultural identity is based primarily on blood quantum and to move away from notions of one person being “more Hawaiian” than another (Ledward, 2007). As illustrated by these examples, it is possible that some laypeople may implicitly believe that folk biology, such as ancestry or blood, is consequential for the maintenance of culture, even while explicitly endorsing culture as a socialized process.^4^

We expect that few laypeople will explicitly describe culture as deterministically biological or rooted in genes. Even those who use DNA services are aware that culture is not completely determined by genes. Yet laypeople may express disappointment and discomfort when discovering a lack of DNA evidence to affirm a current cultural identity (Marcon et al., 2021). These real-world examples demonstrate that people may, at some level, be concerned about folk biology when talking about issues surrounding culture.

## Suggestions for Reducing Lay Misperceptions of Culture as Biological

For researchers who study culture, what unique problems might they face when communicating their results? It is possible that laypeople may consume research about culture, assume that the research is about race, and then misinterpret cultural findings as related to folk biology. For instance, an audience might assume that findings of Americans and Japanese having different visual attention patterns mean that these cultural differences boil down to racial differences, which are then misinterpreted as originating from biological differences. This can be especially apparent when studying psychological processes that are readily associated with neurobiological systems (e.g., visual system). Researchers who study culture, therefore, should be aware that their findings could be misinterpreted. In this section, we provide three suggestions for researchers who want to incorporate culture in their work that can encourage an accurate understanding of cultural research findings for their audience. First, we suggest researchers should explicitly acknowledge whether biological processes are, or not, related to cultural findings in their work. Second, we suggest that researchers carefully and critically consider how the methods they use in their work could lead to different assumptions about culture, some of which might encourage a lay audience to perceive cultural findings as related to folk biology. Third, we suggest that all researchers, whether their work focuses on culture or not, represent all humans as cultural to discourage lay perceptions of culture as related only to people who are non-White/non-Western.

### Explicitly address biological processes

It may be easy for laypeople to misinterpret research about culture as related to folk biology because research about culture often does not mention a link to biology or the natural sciences at all. Indeed, other researchers have noted that the study of culture, overall, has been discrete from the study of natural sciences. As an example, Faulkner and colleagues (2006) analyzed 313 different definitions of culture, all of which came from traditionally nonnatural science disciplines, such as communications or political science, and none from more traditional natural science disciplines. There are likely important historical reasons why; the role of biology in discussions about culture may trigger unpleasant associations of a time when scientists used biological arguments to advance agendas of racial superiority (i.e., *scientific racism*; Jackson & Weidman, 2005), which were gravely problematic and had serious societal implications (Dennis, 1995). By not addressing how biology is, or is not, relevant for the study of culture, researchers may be leaving their audiences to instead rely on lay intuitions about the role of biology for understanding culture.

A way forward for cultural psychology may involve situating culture within nature rather than separate from it (e.g., Kashima, 2000, 2016, 2019). Primatologist Franz De Waal (2001), for instance, argued that culture and nature cannot be easily separated, emphasizing that culture develops within the constraints of biology. This perspective has been incorporated in developmental research because cultural learning relies on biologically prepared capabilities that unfold over the course of development (Keller, 2008). In cultural neuroscience, there are also proposed frameworks attempting to explain how culture and genes complexly interact to predict different psychological outcomes (Kim & Sasaki, 2014) via processes of dual inheritance or gene-culture coevolution across both evolutionary time (Beja-Pereira et al., 2003; Boyd & Richerson, 1985; Feldman & Laland, 1996) and across the life span (Hallmark et al., 2019; Morgan & Rose, 2005). The reality is that the study of culture, broadly, may be incomplete without incorporating biologically informed research.

Our first suggestion is thus directed toward researchers who study culture. One way to increase more accurate, nuanced interpretations of cultural-psychological findings may be to explicitly theorize about the extent to which biological processes, such as biodevelopmental pathways or gene-culture coevolution, are, or are not, relevant for interpreting a specific cultural finding. Although combating implicit beliefs can be difficult in any domain (Greenwald & Lai, 2020), bringing awareness to the issue is an important first step to try to dispel inaccurate assumptions (Sabin, 2022). By explicitly addressing the relevance or irrelevance of biological processes for interpreting cultural findings, lay audiences may be less likely to fill in the gaps themselves by assuming that culture is determined by folk biology, using either crude or inaccurate lay theories. We anticipate that many cultural researchers are primarily interested in studying and theorizing about cultural variation that results from differences in social learning or socialization, and not necessarily about how culture interfaces with biological processes. Yet it would still be valuable to explicitly acknowledge for the audience the extent to which biology may or may not play a role, while at the same time highlighting why the social aspects of culture are important for the current research.

Importantly, it is possible to acknowledge culture and biology as complexly interacting and mutually influencing each other over time without confounding them as the same concept.^5^ Culture does not boil down to biology or vice versa. Within the complex web of cultural and biological processes, it is possible to zoom in on one process or another and tell that small part of the story. We simply argue that researchers studying culture may hold a responsibility to explicitly situate their investigation within this complex web, zooming out and discussing where their findings fit within the larger story, to reduce misinterpretations of cultural findings.

### Consider multiple methods

Another crucial step toward reducing misinterpretations of cultural findings is to anticipate how certain methods may lead lay audiences astray. Common methods used to study culture include but are not limited to (a) demographics as a proxy for culture, (b) self-reported knowledge (cultural identity, beliefs, and values), (c) behaviors or behavioral tendencies on cultural tasks, and (d) cultural priming (for an overview of methods used to study culture, see Table 1). Different methods may be best suited to studying certain cultural phenomena, and therefore a mismatch between methods and cultural phenomena may invite unwanted interpretations. Researchers should be mindful of the implicit assumptions behind certain methods and how lay audiences might interpret findings about culture as being more tied to socialization or biology from specific methods.

**Table 1.** Advantages and Disadvantages of Methods for Studying Culture

Method	Advantages	Disadvantages	Examples
Demographics as a proxy for culture	Straightforward sample-size planning; can be a reliable proxy for cultural values; may capture cultural variation that does not rely on explicit knowledge or awareness of cultural values (e.g., implicitly held cultural values); useful for initial studies of cultural variation because it can capture cultural variation that was not possible to detect via other methods	May inadvertently highlight ancestry as relevant to the research question; does not capture underlying mechanisms because demographics are not psychological variables; may obscure heterogeneity within demographic groups and overemphasize differences between demographic groups	Investigating social-attention differences between European American and East Asian participants (A. S. Cohen et al., 2017)
Self-reported knowledge: cultural identity, values, and beliefs	Opens participant recruitment to many cultures; directly measures psychological mechanism; suitable for capturing explicitly held cultural knowledge that people can accurately report	Not suitable for capturing implicitly held cultural knowledge; may not be accessible or reportable at the individual level if the cultural knowledge is collectively upheld at the societal level; may be interpreted differently across cultural groups because of construct validity, measurement equivalence, and subjective measurement issues	Measuring Asian values as a predictor of emotion suppression in an ethnically diverse American sample (Butler et al., 2007)
Behaviors or behavioral tendencies on cultural tasks	Opens participant recruitment to many cultures; directly measures psychological mechanisms; suitable for capturing both explicitly and implicitly held cultural knowledge; bypasses some cross-cultural measurement issues associated with self-report knowledge method	Not all cultural knowledge can be measured with responses on a behavioral task; may not be clear what the task is measuring (construct validity)	Drawing social networks and comparing size of the circles for self vs. friends to measure independence in European Americans, Western Europeans, and East Asians (Kitayama et al., 2009)
Cultural priming	Opens participant recruitment to many cultures; suitable for manipulating salience of implicitly held cultural knowledge; allows causal interpretations from experimental manipulation of a cultural construct	Theoretical uncertainty about the mechanisms underlying certain priming effects in different psychological domains; uncertain whether different cultural groups respond to priming in the same manner	Priming independent and interdependent self-construal to influence neural activity for European American monoculturals and Asian American biculturals (Fong et al., 2014)

#### Demographics as a proxy for culture

##### Advantages

Using demographic information, often for ethnicity or nationality, is one of the most common methods used to study culture and comes with a few advantages. First, it is a practical method for attaining cultural variability in a sample because researchers can easily recruit balanced numbers of participants from different cultural groups. Because researchers often need to consider many additional factors in a cultural study, the demographics method may save time and resources by making it simpler to plan target sample sizes.

Second, it can sometimes be valid to assume that ethnicities or nationalities of participants may covary enough with meaningful average differences in learned values or behaviors between groups based on past research in the field. For example, national boundaries can at times delineate consistent and reliable cultural contexts bound by national policies, norms, and practices that justify meaningful study at the country-level aggregate (Akaliyski et al., 2021; Minkov & Hofstede, 2012; Smith et al., 2002; however, see also van Pinxteren, 2020). This advantage is best yielded from a multiple-group comparison approach (e.g., comparing more than two countries) because it may be more likely to encourage a psychological perception of cultural variation as continuous compared with a two-group comparison approach (e.g., United States vs. Japan), which might encourage a more dichotomous perception of culture. Depending on one’s research question, it can certainly be reasonable to utilize the demographics method, especially when previous studies have found reliable support using demographic variables.

Third, the demographics method may also capture cultural effects that are implicit. Participants can be unaware of how previous exposure to a system of cultural knowledge can shape their current implicit processes, behaviors, or attitudes, and thus they may be unable to accurately report on this process. This is especially true for psychological processes that they cannot explain or control, such as automatic processes.

Fourth, preplanning data collection from demographic groups that are exposed to different cultural knowledge may capture meaningful cultural differences that were not possible to detect via other measures available at that time. Indeed, many investigations in cultural psychology begin by using this method, and then down the line glimpses of an underlying explanation become clearer with later methodological advances, such as measuring cultural differences in neural activity (Zhu et al., 2007). This is especially useful if the goal for the research is to describe or explore whether cultural variation exists for psychological domains for which there is limited understanding or theorizing at the time about how culture plays a role.

##### Disadvantages

Despite the advantages, the demographics method has many disadvantages. These disadvantages are particularly salient when using the two-group comparison, and especially so when using the overused East versus West or the U.S. versus non-U.S. comparisons. Our stance is that including a greater number of cultural groups, particularly those underrepresented in psychological research, will advance our understanding of culture more so than just two. We would suggest considering the demographics method as a starting point for new investigations but not an end in itself because of the clear theoretical limitations of this method.

First, the demographics method may unintentionally point to ancestry, rather than cultural experiences, as relevant to the research question at hand. Although technically participants could interpret the question “What is your cultural background?” as a question about learned cultural experiences, participants tend to understand and subsequently answer the question as one about family ancestry (for evidence of the perceived link between race and ancestry in American participants, e.g., see J. M. Chen et al., 2018). As an example, a third-generation American of Mexican heritage may feel more culturally American than Mexican, but they may choose “Latino/a/x” or write “Mexican” specifically in response to a question about their culture, indicating their ancestry. Using the demographics method to studying culture may thus inadvertently not only lead participants to report on their ancestry but also mislead lay audiences to think of culture as related to ancestry. Yet researchers typically do not intend to conceptualize ancestry as a potential cause for differences in their outcome of interest; rather, they are often examining learned cultural practices as the cause. Using the demographics method may thus not be the ideal way to test a research question about learned cultural practices because it relies on comparing different racial or ethnic groups, which vary not only in learned cultural practices but also potentially in ancestry. Even when using large-scale, multination investigations, it is important to note that the major disadvantage of the demographics method still applies: Demographics may inadvertently highlight ancestry as important for studying culture.

Second, the demographics method may lead to the misinterpretation that people embody psychological aspects of their culture simply because of their demographic categorization (Quintana et al., 2001), promoting cultural stereotyping (Matsumoto, 1999). However, demographic categories are not psychological variables (Betancourt & López, 1993; Hermans & Kempen, 1998; Okazaki & Sue, 1995; Quintana et al., 2001; Sue, 1999). The main disadvantage of the demographics method is that it often does not provide clear underlying explanations, including basic psychological mechanisms and socioecological dynamics shaping any downstream tendencies evident in cultural comparisons (Matsumoto, 1999). Even if it is the case that comparing cultural groups measured by demographic variables can produce robust effects, there is still something to be explained at the level of psychology as to *why* those effects occur. Thus, an undeniable downside of the demographics method is that, ultimately, a finding of cultural differences between demographic categories is unsatisfying because it lacks psychological explanation.

Last, using the demographics method can unintentionally ignore meaningful differences within a demographic group and obscure meaningful similarities between demographic groups. All nations or ethnicities have some degree of heterogeneity, and by focusing on differences between groups, meaningful cultural variation that exists within groups may be easily overlooked (Lalonde et al., 2013). Just as within-group differences are easy to ignore, between-group differences are hard to resist with the demographics method (Lalonde et al., 2015). This can be problematic because cultural psychology is relevant to understanding not only domains for which cultures differ but also domains for which they are similar (Causadias, Korous, & Cahill, 2018; Lalonde et al., 2015; Wang, 2016). The demographics method may inadvertently lead the audience to generalize groups of people within those demographic categories, which may have social implications for those cultural groups.

In sum, although relying on demographics as a proxy for culture can at times be useful, researchers should carefully consider how their results may be interpreted based on this study method. Compared with other methods, the demographics method is, by far, more likely to encourage assumptions that culture is related to ancestry and subsequent generalizations of people from different cultural groups. The solution is not to abandon the method entirely but rather to discuss clear limitations and consider alternative or a combination of methods, such as those described below, that could more effectively address the research question at hand.

#### Self-reported knowledge: cultural identity, beliefs, and values

##### Advantages

Researchers may study culture as learned knowledge by asking participants to self-report on more *explicit* forms of culture, including certain aspects of their identity (e.g., Bicultural Identification Integration Scale; Benet-Martínez et al., 2002), beliefs (e.g., Analysis-Holism Scale; I. Choi et al., 2007), and values (e.g., Individualism-Collectivism Scale: Triandis & Gelfand, 1998; Self-Construal Scale: Singelis, 1994). Although relatively less common compared with using the demographics method, the self-reported knowledge method comes with certain advantages. First, studies using this method are not restricted to members of a certain demographic variable and could be theoretically open to anyone, which simplifies data collection. As an example, Butler and colleagues (2007) used endorsement of “Asian values” across multiple ethnic groups as a measure of culture, opening up data collection to diverse groups. More specifically, a European American or African American participant in their study could be considered culturally “Asian” if they strongly endorsed Asian values on an explicit self-reported value measure. This method puts the cultural variable as the focus, as opposed to any specific cultural group. A second advantage of the self-reported knowledge method is that it allows culture to be measured directly as a psychological mechanism that could be used to explain group-level differences (e.g., Gelfand et al., 2011). Third, this method is well matched with researchers’ definitions of culture that emphasize socialized components because self-report knowledge methods assume participants can report on cultural values that they explicitly hold.

##### Disadvantages

There are several limitations, however, to the self-reported knowledge method. First, people may not always be aware of the cultural knowledge they hold (Nisbett & Wilson, 1977). Thus, this method may not adequately measure implicit cultural knowledge that people cannot report on.

Second, cultural knowledge tends to be maintained collectively in a way that goes beyond the individual. For example, even when an individual is not personally involved in an occupation that might directly promote interdependent values (e.g., farming), they may still hold those cultural values (Uchida et al., 2019; Uskul et al., 2008)—a cultural difference identifiable by their categorical belonging in a larger farming community (i.e., by using the demographics method) but not necessarily by an individual’s self-reported personal identification with those economic activities. Relatedly, theoretical and empirical research suggests that cultural differences cannot always be reduced to individual differences (Na et al., 2010). As an example, independent and interdependent self-construal had long been thought to be the hypothesized mechanism explaining observed cultural differences on several psychological domains (Markus & Kitayama, 1991). However, researchers have previously documented that there are not always cultural differences when measuring independent or interdependent self-construal using self-report methods, and when there are differences, they are sometimes not in the predicted direction (Matsumoto, 1999; for related criticism of individualism-collectivism, see also Oyserman et al., 2002). Thus, cultural differences at the group level may not be reflected in individual-level self-report methods.

Third, there are methodological issues with measuring self-report knowledge across cultural groups. There may be construct-validity issues when trying to measure the same construct across different cultural groups because the meaning of the construct may not be equivalent or comparable. For example, the meaning of interdependence may differ between European Canadians and East Asians (Ng et al., 2015; see also Peng et al., 1997). In the earlier example given from Butler and colleagues (2007), a valid question is whether “Asian values” have the same meaning for a European American participant as it does for an Asian participant. Self-report knowledge methods are also subject to potential measurement equivalence problems from translated measures. Even when using translation and back-translation methods between languages, there are meanings that do not translate well. Finally, there are known subjective measurement issues associated with between-culture comparisons, such as the reference group effect, which shows that cultural differences can be obscured when different groups are using different reference groups to evaluate their own beliefs or behaviors (Heine et al., 2001).

In sum, the self-reported knowledge method may be most appropriate for testing theories of learned explicit cultural knowledge, relatively independent of biologically based influences, but it may fall short of capturing implicit cultural knowledge, and it can be a challenge to make sure that self-reported knowledge is equivalent or comparable across cultural contexts.

#### Behaviors or behavioral tendencies on cultural tasks

##### Advantages

Measuring behaviors or behavioral tendencies on *cultural tasks*, or scripted procedures that act as means to maintain cultural ideals, is a particularly useful way to capture more implicit aspects of culture (Kitayama et al., 2009). One example of a cultural task is the symbolic self-inflation task, which instructs participants to draw circles representing themselves and friends in their social networks. The relatively larger size of the circles for the self versus their friends is used to implicitly measure independence (Kitayama et al., 2009). Like the self-reported knowledge method, an advantage of the cultural-task method is that it opens participant recruitment beyond specific cultural groups. Another advantage shared with the self-reported knowledge method is that the cultural-task method can directly measure potential psychological mechanisms. Importantly, a unique advantage of the cultural-task method is that it may access *implicit* cultural knowledge that the self-reported knowledge method cannot. The cultural-task method can also avoid the pitfalls of the self-report knowledge method when it comes to measurement issues surrounding questionnaire use (e.g., translation issues, reference-group effect). In this way, measuring culture via cultural tasks may have the same benefits as the self-reported knowledge method but can potentially capture both explicitly and implicitly held cultural knowledge.

##### Disadvantages

One obvious downside of the cultural-task method is that some domains of cultural knowledge cannot be easily measured with observable behaviors, or that it can be difficult to design a cultural task to measure certain constructs. Another potential issue is that for some cultural tasks, it may not be clear what the task is measuring conceptually. As with any measure, construct validity is important to consider with cultural tasks, particularly for those that measure constructs implicitly.

#### Cultural priming

Methods that can address issues of implicit cultural influence include cultural-priming tasks. Research using cultural information as a prime assumes that culture is learned via a network of associations, and making salient one aspect of culture (e.g., language) can subsequently activate associated cultural knowledge or behaviors (e.g., cognitive style; S. X. Chen, 2015). Research on self-construal priming (Gardner et al., 1999) and frame switching (Hong et al., 2000) has shown that priming features of a culture that are learned, such as the concept of independent versus interdependent self-construals (e.g., circling “I” or “we” pronouns) or cultural icons that broadly represent a culture (e.g., the Great Wall of China or the Statue of Liberty), can influence psychological tendencies associated with the respective cultures.

##### Advantages

Priming methods can open the potential pool of participants beyond specific cultural samples, like the self-reported knowledge and cultural task methods. For example, because independent and interdependent self-construal are theorized to coexist in varying degrees for any individual, anyone could theoretically be primed to demonstrate the effects of independent and interdependent self-construal on various behaviors (Oyserman & Lee, 2008). The rationale behind priming is based primarily on learned exposure, suggesting that cultural priming may be best suited to test theories of implicit cultural influence. Unlike correlational or quasi-experimental methods, priming methods involve experimental manipulation, allowing inferences about causality. It is possible to manipulate the proposed psychological mechanism linking culture and an outcome of interest by simply priming participants with a task designed to activate the psychological mechanism.

##### Disadvantages

The disadvantages of priming, however, include lack of clarity about exactly *how* priming can lead to differences on various psychological domains. For example, priming interdependent self-construal has been observed to increase visual attention to the context in a scene (e.g., airport landing strip; H. Choi et al., 2016) but produces mixed findings when the context is social (e.g., multiple eye gazes; Lo et al., 2021). The same priming task may also influence different cultural groups in different ways, given that different groups may have access to different cultural knowledge (e.g., monoculturals and biculturals; Fong et al., 2014; Lo et al., 2021). Theorizing about priming may also need to incorporate a more integrated biocultural approach to be fully understood. For example, it is possible that people are differentially sensitive to cultural priming depending on age (for developmental reasons) and cognitive domain (for evolutionary reasons). With more developed theorizing about how and why cultural primes can lead to associated thoughts and behaviors, priming methods could eventually have greater potential to examine research questions that incorporate both learned and biological aspects of culture.

#### Complementarity of methods

These different methods for studying culture can be complementary to each other, allowing researchers to take advantage of the pros associated with each. For example, using the demographics method for recruiting participants from many different nations, in combination with self-report knowledge and behavioral-task methods, allows for sufficient cultural variation (i.e., from the multination demographics method) and measurement for psychological mechanisms (i.e., from self-report measures and behavioral tasks). Each method may also capture different, although at times partially overlapping, aspects of culture. Both self-report knowledge and priming methods, for instance, assume some degree of learned cultural knowledge, either explicit or implicit. Specifically, the self-report knowledge method mainly captures explicit knowledge, whereas priming methods are suitable for capturing implicit cultural knowledge as well. However, it is also important to consider whether the assumptions underlying each method may change when used together. There are possible measurement issues that arise when combining the self-report knowledge and demographics methods to recruit different cultural groups. For example, different cultural groups may have separate reference groups in mind when answering self-report questionnaires. It is important to thoughtfully consider how certain combinations of methods may create unique advantages and disadvantages for studying culture.

### Represent all people as cultural

Cultural-psychological research tends to be synonymous with research about people who are ethnic or racial minorities (commonly, non-White individuals in Western cultural contexts) or people from non-Western cultural contexts (A. B. Cohen, 2009). Given that ethnic or racial minorities and non-Western cultural contexts are highly underrepresented in psychological research, more research using underrepresented groups may encourage critical discussions about the “typically invisible gaps” in the field, which tends to be heavily based on Western cultural experiences (Adams, 2005, p. 965).

An unintended consequence of cultural research being synonymous with research about ethnic or racial minorities or non-Western cultural contexts, however, is that this may be contributing to the implicit message that psychological research with White or Western samples pertains to universal phenomena that are not culturally influenced (cf. Causadias, Vitriol, & Atkin, 2018; Cheon et al., 2020; Roberts et al., 2020). At the same time, an emphasis on studying non-White/non-Western samples in cultural psychology may inadvertently lead readers (especially lay audiences from Western societies) to believe that culture is relevant only for non-White/non-Western samples. These findings expose a *cultural misattribution bias* (Causadias, Vitriol, & Atkin, 2018) within psychology that White individuals in the United States or other Western countries are considered the default and assumed to reflect universal characteristics, whereas people from minority or non-Western samples are “cultural” (Adams et al., 2015; Cheon et al., 2020). Recent archival research is consistent with this possibility. Psychology articles using samples outside the United States were more likely to specify the sample’s characteristics (e.g., as Indian or Brazilian) in their titles compared with articles using American samples (Cheon et al., 2020). Studies focusing on culture tend to have samples with more non-White/non-Western participants than studies that do not focus on culture, and researchers were more likely to favor cultural explanations for results about non-White/non-Western samples but favor psychological explanations for results about White/Western samples (Causadias, Vitriol, & Atkin, 2018). Studies conducted in non-Western cultural contexts may also be perceived as less important, interesting, or relevant, suggesting there are also particularly inequitable editorial standards for non-White/non-Western researchers who conduct research in non-White/non-Western cultural contexts (Bou Zeinddeine et al., 2022; Roberts et al., 2020). Our own perspective is that this existing bias points to systemic power structures in academic research (see Roberts et al., 2020) that maintain inaccurate views of what counts as “general psychological research” with seemingly little cultural or contextual constraint that is worth publishing in top-tier general psychology journals versus “cultural research” that is considered niche and relevant for more specialized cultural journals.

We argue that the cultural misattribution bias may also perpetuate the culture-biology link by encouraging lay audiences to misinterpret research about culture as only relevant to non-White/non-Western individuals (i.e., reinforcing the culture-race link). This can then lead to downstream associations between race and folk biology (e.g., ancestry). The reality is that all people sampled in psychological research are shaped by multiple forms of culture (e.g., nationality, religion, social class; A. B. Cohen, 2009) that produce the cultural environment around them (Shweder, 1995). That is, all people are inherently cultural beings, and it is important to communicate this to lay audiences. To guide lay audiences’ assumptions away from the idea that culture is only about groups that have non-Western ancestry, we offer two suggestions directed toward cultural and noncultural researchers to remind laypeople that everyone is shaped by multiple forms of culture.

#### Study multiple forms of cultures

The first suggestion is most relevant for cultural researchers. We suggest cultural researchers carefully consider whether a chosen cultural context is appropriate for a given research question. It may seem simplest to go with previously studied cultural contexts, which often encompass cross-national or cross-ethnic comparisons, especially between Eastern and Western cultures. There are sometimes good reasons for choosing commonly used cultural contexts, such as basing hypotheses on previous findings with similar cultural contexts. However, as emphasized by A. B. Cohen (2009), culture is not synonymous with nationality or ethnicity. We should consider whether other forms of culture may be more appropriate for our research questions to discourage the idea that the term “culture” only refers to “foreign” cultural groups. For example, region and ecological practices (e.g., Southern region of the United States; D. Cohen & Nisbett, 1997; Nisbett & Cohen, 1996), religion (A. B. Cohen & Rozin, 2001; Johnson et al., 2011), and social class (Fiske & Markus, 2012; Grossmann & Varnum, 2011; Stephens et al., 2007) are all forms of culture, and it may make more sense to select these forms of culture over nationality or ethnicity for certain research questions. For example, those interested in how culture shapes prosocial behavior may want to carefully consider religion in addition to nationality or ethnicity, given that prosociality is explicitly tied to many different religious teachings.

It may also be helpful for cultural researchers to consider integrating many forms of culture in their work given that people are influenced by multiple forms of culture simultaneously. Some forms of culture may be more influential or relevant for a particular psychological process than others, and on top of that, the different forms of culture may interact to produce psychological variation. For example, nation-level cultural values and religion may interact to produce variation in well-being: Religious individuals who perceive religiosity as an accepted social norm tend to have higher well-being, but only if they live in culturally tight nations (Pearson et al., 2022). It is important to recognize that other forms of culture in addition to nationality or ethnicity can be highly influential on psychological functioning. The goal is to encourage broader use of the term “culture” in research to refer to other forms of culture more equitably.

#### Consider all psychological research as cultural

Our second suggestion is directed toward all researchers. We argue for contextualizing research as specific to the cultural context equitably, and not only for research on non-Western/non-White samples. This suggestion is not new; culturally contextualizing all psychological research encourages accurate perceptions that most research findings are not broadly generalizable or universal (e.g., Cheon et al., 2020; Rad et al., 2018). Research that attempts to explain psychological universals should be effortful and theoretically guided, demonstrating invariance across cultural contexts that vary along relevant dimensions (for a comment, see Majid, 2023; for an example investigation of odor pleasantness across cultures, see Arshamian et al., 2022). In line with recommendations in the current article, contextualizing all research as cultural can have the added benefit of promoting a conceptualization of culture that is not exclusively associated with non-Western/non-White individuals and folk biology (e.g., ancestry and genes). Below, we provide examples of where researchers can contextualize their work as cultural:

1. Abstract or title: Labeling where the research was conducted and who the participants were in the abstract or title can serve as a clear reminder that all research about people is cultural (Cheon et al., 2020; Rad et al., 2018). If Study A was conducted in a large university in the U.S. Northeast, with a largely Christian population, and Study B was conducted in a large university in the northwest region of India with a largely Sikh and Hindu population, then both studies should be fairly labeled as such to indicate to lay audiences that findings from both studies are specific to certain cultural contexts. This equity is important, because archival research suggests that the sample’s country is included less often in study titles for samples from the global North (i.e., Europe, North America, Australia, New Zealand; Torres & Alburez-Gutierrez, 2022) and Western, educated, industrialized, rich, and democratic (WEIRD) countries (i.e., Henrich et al., 2010). In contrast, studies with samples from the global South and non-WEIRD countries include the sample’s country more often in study titles, which subsequently receive less scientific attention (e.g., citations) than studies from the global North or WEIRD countries (Kahalon et al., 2022; Torres & Alburez-Gutierrez, 2022).
2. Introduction: Culturally contextualize the theoretical framework and overview of past literature. Where were most of the studies conducted, and where was the theoretical framework developed? This exercise can help researchers realize where most of the research in their field comes from (i.e., oftentimes North America or Europe) and encourages researchers to perceive their research as culturally bound until there is more globally conducted research and evidence.
3. Methods/results: Articles should report the sample breakdown of ethnic/racial groups, religions, social classes, and regions (e.g., U.S. Midwest, U.S. South), all of which may help culturally contextualize the sample equitably (Rad et al., 2018; Roberts et al., 2020).

We hope that our suggestion to represent *all* people as cultural—by studying multiple forms of culture and contextualizing research equitably—may encourage more accurate lay perceptions of culture.

## How to Integrate Suggestions

You cannot study a fish without considering water an important part of its life. Likewise, you cannot fully understand human psychology without studying the cultural contexts that humans live in. This is the general idea that scientists who study culture intend to convey in their research. It is unfortunate, then, that laypeople (and scientists) may misinterpret and overgeneralize cultural psychological findings that are taught in courses or diversity interventions as cultural stereotyping (Buchtel, 2014; Shepherd, 2019), a finding that should be alarming for all psychologists. The reality is that cultural research is consumed by people with different preconceptions of what culture is, and with varying understandings of the relationship between culture and biology. It is therefore important for any researcher to be explicit about how biology is—or is not—relevant for their research questions, to carefully consider the assumptions that come with their methodological choices, and to encourage a perception of all research as culturally bounded.

Below, we provide an example to illustrate how researchers can integrate our suggestions. For instance, consider a cultural researcher who wants to study how cultural socialization shapes visual attention to the behaviors of strangers, and they want to use the demographics method to collect participants from the United States and Japan. This researcher hypothesizes that Japan has a less relationally mobile culture (i.e., interpersonal networks are less flexible) than the United States, and that people from Japan are therefore more context-sensitive in their attention toward strangers than people from the United States (San Martin et al., 2019). They then use the demographics method to measure culture and subsequently realize that observing cultural differences on a cognitive task alone may be misperceived as observing biological differences, especially because cognitive processes are perceived to be strongly driven by real neurobiological processes (e.g., visual system). What should they do to reduce reader misinterpretations?

1. Explicitly address biological processes. The researcher should take a step back and think carefully about whether the role of culture may be tied with real biological processes, such as the biodevelopmental pathway that allows for the socialization of different attention patterns to develop. In this case, the researcher may decide that this biodevelopmental pathway, although important, is beyond the scope for them to explicitly address in their research methodology. They acknowledge in their introduction section that there are real biological processes necessary for the visual attention system to develop and be calibrated by sociocultural experiences. At the same time, they emphasize that the aim of the study is to measure or manipulate the effect of sociocultural experiences. It may be important to explicitly theorize about the origins of cultural differences in the psychological phenomenon of interest, or else laypeople may misinterpret cultural differences in psychology to be indicative of differences in folk biology (e.g., ancestry).
2. Consider multiple methods. To minimize misperceptions of cultural differences as biological differences, the researcher may implement methodological changes such as measuring the hypothesized psychological mechanism directly (e.g., relational mobility), in addition to, or instead of, using the demographics method. This methodological change would support their goal of testing cultural differences in visual attention as socialized (in this case, resulting from differences in nation-level relational mobility). The researcher should also consider theorizing whether relational mobility can be captured as explicit (i.e., self-report method) or implicit (i.e., behavior task method) cultural knowledge. Given the pitfalls associated with self-report methods alone (Matsumoto, 1999), one strategy would be to incorporate both self-report and behavioral-task methods. Another strategy would be to use priming methods to directly demonstrate the causal role of relational mobility on visual attention (San Martin et al., 2019).
3. Represent all people as cultural. If the researcher is interested in directly extending previous North America versus Japan findings in visual attention, they may feel justified in selecting these nations as their cultural context. They should then aim to provide a breakdown of each group to contextualize both samples (e.g., social class, region). However, if they were interested in demonstrating that relational mobility is what matters for shaping visual attention strategies, they may consider selecting other forms of culture that are less likely to be associated with folk biology and can demonstrate variation in relational mobility, such as social class. Finally, to accurately contextualize their research, they should consider clearly labeling their sample in the abstract or title to promote accurate interpretations of their research as occurring within a specific cultural context. The decision to label should be made uniformly, whether or not their sample comes from a WEIRD cultural context.

By carefully considering these suggestions to explicitly address biological processes, consider multiple methods, and represent all people as cultural, researchers can reduce misinterpretations of their findings while contributing to the broader picture about how culture shapes psychology.

## Conclusion

We provide these suggestions in hopes that researchers—whether they directly study culture or not—can play an active role in disseminating their work accurately and effectively to broader audiences. We hope that researchers will carefully consider the assumptions lay audiences make when consuming their research and act accordingly to promote more accurate interpretations. If researchers can explicitly communicate about the role of biology in their cultural research, the assumptions of different methods, and the fact that all people are cultural, this can help lay audiences to more accurately understand whether and how different aspects of culture play a role in psychological processes.

## Transparency

*Action Editor:* Leonel Garcia-Marques

*Editor:* Interim Editorial Panel

## Notes

1. Multiple intuitions about culture exist in the human mind. Here we highlight intuitions about culture as biological because it is mismatched with common scientific definitions of culture as learned.

2. Although we write primarily from our perspective as cultural psychologists, this problem is relevant in any field that incorporates culture in their work.

3. Kānaka is used to refer to a countable plural, whereas Kanaka (without the kahakō or macron) refers to the singular.

4. Notable exceptions exist because for some cultural groups it is more explicitly believed that membership into the cultural group is biological. For example, in more traditional forms of Judaism, it is an explicit belief that Jewish heritage is biologically passed down through the maternal line (A. B. Cohen, 2009), in addition to coming from socialized factors.

5. We would like to thank one of our anonymous reviewers for helping us clarify this point.
